# Stabilization of Memory States by Stochastic Facilitating Synapses

**DOI:** 10.1186/2190-8567-3-19

**Published:** 2013-12-06

**Authors:** Paul Miller

**Affiliations:** 1 Volen National Center for Complex Systems and Department of Biology, Brandeis University, 415 South Street, Waltham, MA, 02454, USA

**Keywords:** Dynamic synapses, Stochastic processes, Facilitation, Poisson process, Bistability, Persistent activity, Short-term memory, First-passage time

## Abstract

Bistability within a small neural circuit can arise through an appropriate strength of excitatory recurrent feedback. The stability of a state of neural activity, measured by the mean dwelling time before a noise-induced transition to another state, depends on the neural firing-rate curves, the net strength of excitatory feedback, the statistics of spike times, and increases exponentially with the number of equivalent neurons in the circuit. Here, we show that such stability is greatly enhanced by synaptic facilitation and reduced by synaptic depression. We take into account the alteration in times of synaptic vesicle release, by calculating distributions of inter-release intervals of a synapse, which differ from the distribution of its incoming interspike intervals when the synapse is dynamic. In particular, release intervals produced by a Poisson spike train have a coefficient of variation greater than one when synapses are probabilistic and facilitating, whereas the coefficient of variation is less than one when synapses are depressing. However, in spite of the increased variability in postsynaptic input produced by facilitating synapses, their dominant effect is reduced synaptic efficacy at low input rates compared to high rates, which increases the curvature of neural input-output functions, leading to wider regions of bistability in parameter space and enhanced lifetimes of memory states. Our results are based on analytic methods with approximate formulae and bolstered by simulations of both Poisson processes and of circuits of noisy spiking model neurons.

## 1 Introduction

Circuits of reciprocally connected neurons have been long considered as a basis for the maintenance of persistent activity [1]. Such persistent neuronal firing that continues for many seconds after a transient input can represent a short-term memory of prior stimuli [2]. Indeed, Hebb’s famous postulate [3] that causally correlated firing of connected neurons could lead to a strengthening of the connection, was based on the suggestion that the correlated firing would be maintained in a recurrently connected cell assembly beyond the time of a transient stimulus [3]. Since then, analytic and computational models have demonstrated the ability of such recurrent networks to produce multiple discrete attractor states [4], as in Hopfield networks [5,6], or to be capable of integration over time via a marginally stable network, often termed a line attractor [7,8]. Much of the work on these systems has assumed either static synapses, or considered changes in synaptic strength via long-term plasticity occurring on a much slower timescale than the dynamics of neuronal responses. Here, we add some new results pertaining to the less well-studied effects of short-term plasticity—changes in synaptic strength that arise on a timescale of seconds, the same timescale as that of persistent activity—within recurrent discrete attractor networks. 

The two forms of short-term synaptic plasticity—facilitation and depression—affect all synapses of the presynaptic cell according to its train of action potentials. Synaptic facilitation refers to a temporary enhancement of synaptic efficacy in the few hundreds of milliseconds following each spike, effectively strengthening connections to postsynaptic cells as presynaptic firing rate increases. Synaptic depression is the opposite effect—reduced synaptic efficacy in the few hundreds of milliseconds following a presynaptic spike, effectively weakening connection strengths as presynaptic firing rate increases. The dynamics of these processes (Table 1) also impacts the variability in postsynaptic conductance, in particular when synaptic transmission is treated as a stochastic event. The variability affects information processing via the signal-to-noise ratio [9-11] and also determines the stability, or robustness, of discrete memory states [12,13]. 

**Table 1:** Stochastic synapse model and parameters. (S) for single-synapse model; (M) for memory model calculations, where different

(a) Presynaptic components	
Synapse	Determinant of release probability, $P_{\mathrm{rel}}(t)$
Static	$P_{\mathrm{rel}}(t)=p_{0}$
Facilitating	$P_{\mathrm{rel}}(t)=p_{0}F(t)$, where between spikes: $\tau_{F}\frac{dF}{dt}=(1-F)$ and immediately following each spike: $F^{+}=F^{-}+f_{F}(\frac{1}{p_{0}}-F^{-})$
Depressing	$P_{\mathrm{rel}}(t)=p_{0}V$, where *P*(*V* = 1)=*D*(*t*); *P*(*V* = 0)=1 − *D*(*t*). Between spikes: $\tau_{D}\frac{dD}{dt}=(1-D)$ and immediately following successful release, $D^{+}=0$, while immediately following unsuccessful release, $D^{+}=D^{-}$

(b) Postsynaptic components	
Synapse	Determinant of synaptic gating variable, *s*(*t*)
All	Following successful release: $s^{+}=s^{-}+\tilde{\alpha }(1-s^{-})$
	Between successful releases: $\tau_{s}\frac{ds}{dt}=-s$

(c) Parameters					
Synapse	Presynaptic *τ*	$p_{0}$	Factors	Postsynaptic $\tau_{s}$	$\tilde{\alpha }$
Static	–	0.5 (S)	–	100 ms	1 − exp(−0.25)
		0.5 (M)			
Facilitating	$\tau_{F}=500\;\mathrm{ms}$	0.1 (S)	$f_{F}=0.5$ (S)	100 ms	1 − exp(−0.25)
		0.25 (M)	$f_{F}=0.25$ (M)		
Depressing	$\tau_{D}=250\;\mathrm{ms}$	0.5	–	100 ms	1 − exp(−0.25)

When analyzing the stability of discrete states, we focus on the mean value of and fluctuations within the postsynaptic feedback conductance, since that is the variable with a slow enough time constant to maintain persistent activity in standard models of network-produced memory states [14,15]. In our formalism, we rely on fluctuations in this NMDA receptor-mediated feedback conductance to be on a slower timescale (100 ms) than the membrane time constant, which is short (<10 ms), in part because each cell receives a barrage of balanced excitatory and inhibitory inputs. When synapses are dynamic, both the mean postsynaptic conductance and its fluctuations are altered from the case of static synapses. 

Here, we show how a presynaptic Poisson spike train, which produces an exponential distribution of interspike intervals (ISIs), produces a distribution of inter-release intervals (IRIs) that is not exponential if synapses are either facilitating or depressing. We then consider how the nonexponential distribution of IRIs affects both the mean and standard deviation of the postsynaptic conductance differently from the exponential, Poisson, distribution of IRIs. These results affect the calculation of stability of memory states, yielding differences in the parameter ranges where bistability exists and producing large changes in the spontaneous transition times between states, which limit their stability.

A two-state memory system is limited by the lifetime of the less stable state [16]. For a given system, one can typically vary any parameter so as to enhance the lifetime of one state while reducing the lifetime of the other state. If we define the system’s stability as the lifetime of the less stable state, then the optimal stability of a system arises when the lifetimes of the two states are equal. In this paper, for a given system, defined by the neural firing-rate curve and type of synapse, we parametrically scale the total feedback connection strength to determine the system’s optimal stability. In so doing, we find that optimal stability of bistable neural circuits is enhanced by synaptic facilitation. 

## 2 Statistics of Synaptic Transmission Through Probabilistic Dynamic Synapses

**Fig. 1:**
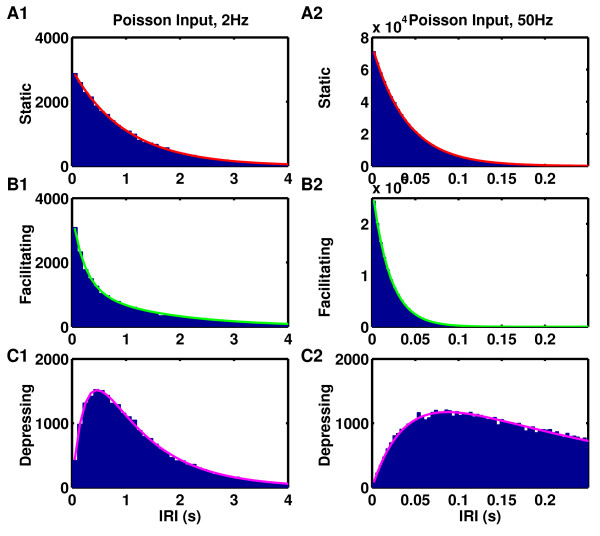
Dynamic synapses alter the distribution of interrelease intervals (IRI) of one vesicle produced by a Poisson spike train. **a1**–**a2** Histogram of IRIs for static synapses is exponential ($p_{0}=0.5$). **b1**–**b2** Histogram of IRIs for a facilitating synapse is more sharply peaked than exponential (with a CV greater than one) ($p_{0}=0.1$, $f_{F}=0.5$, $\tau_{F}=500\text{ ms}$). **c1**–**c2** Histogram of IRIs for a probabilistic depressing synapse has a dip at low intervals (producing a CV less than one) ($p_{0}=0.5$, $\tau_{D}=250\text{ ms}$). **a1**, **b1**, **c1** Presynaptic Poisson spike train of 2 Hz. **a2**, **b2**, **c2** Presynaptic Poisson spike train of 50 Hz

In the following, we assume that synaptic facilitation and depression operate by modifying the release probability of presynaptic vesicles. Following vesicle release, neurotransmitter binds to receptors in the postsynaptic terminal. The fraction of receptors bound at any one time determines the fraction of open channels, known as the gating variable, *s*, which is proportional to the conductance producing current flow into the postsynaptic cell. The dependence of *s* on presynaptic firing is affected by the dynamic properties of the intervening synapse. In particular, the distribution of intervals between vesicle release events is not identical to the interspike interval (ISI) distribution: facilitating synapses increase the likelihood of short inter-release intervals (IRIs) compared to long intervals, so increase the coefficient of variation (CV); whereas depressing synapses make short release intervals unlikely and produce a more regular sequence of release intervals, reducing the CV (Fig. 1). While the means of these distributions can be calculated by standard methods [17], it is valuable to know the full distribution, since changes in the CV of IRIs affect the variability of the postsynaptic conductance, and thus alter properties like signal-to-noise ratio and the stability of memory states to noise fluctuations. 

### 2.1 Distribution of Release Times for a Poisson Spike Train Through Stochastic Depressing Synapses

The distribution of release times of a vesicle for depressing synapses with a single release site is simpler to calculate than that for facilitating synapses, because when considering synaptic depression alone, the probability of release from a single site simply depends on the time since last release of a vesicle from that site. Therefore, we will solve for depressing synapses before moving to the case of facilitating synapses, where the probability of release depends on the number of intervening spikes. The subsequent result for facilitating synapses will prove to be more biologically relevant, as synapses typically contain multiple releasable vesicles, so it is only in the case where the baseline release probability is low—in which case facilitation dominates—that failure of release is common enough to affect the distribution of release times. The case of probabilistic release in depressing synapses with multiple release sites is more complex, though the first two moments of the IRI probability distribution have been calculated by others [18]. 

Synaptic depression arises because of the time needed to recycle and replenish vesicles following release of neurotransmitter. Synaptic depression can be treated stochastically [19] by assuming vesicle recovery is a Poisson process, with the likelihood of a vesicle being release-ready, or “docked,” as $P(V=1)=P_{D}=1-e^{-T/\tau_{D}}$, where *T* is the time since the prior vesicle release. Thus, the distribution of inter-release intervals (IRIs) can be calculated by requiring that a vesicle be docked within the interval and then adding the time for a spike to appear after the vesicle is docked. We assume a docked vesicle has a release probability of $p_{0}$ and incoming spikes arrive as a Poisson process of rate *r*. Since probability of docking between time $T_{D}$ and $T_{D}+\delta T_{D}$ is $\delta T_{D}(dP_{D}/dT)$ evaluated at $T_{D}$, or

(1) $$P(T_{D})\delta T_{D}=\delta T_{D}e^{-T/\tau_{D}}/\tau_{D}$$

and the probability of the first spike after time $T_{D}$ being at time *T* and causing release as $e^{-p_{0}r(T-T_{D})}$, we have

(2) $$P(T)=\int_{0}^{T}\frac{e^{-T_{D}/\tau_{D}}}{\tau_{D}}\cdot e^{-p_{0}r(T-T_{D})}dT_{D}$$

so

(3) $$P(T)=\frac{p_{0}r}{p_{0}r\tau_{D}-1}\left(e^{-T/\tau_{D}}-e^{-p_{0}rT}\right)$$

which leads to a mean IRI of

(4) $$\langle T\rangle =\tau_{D}+\frac{1}{p_{0}r}.$$

The reduction in probability of small IRIs is a simple example of the temporal filtering of information presented by others [18]. The addition of the extra probabilistic process of vesicle recovery, which underlies synaptic depression, causes IRIs to be more regular, as evidenced in Eqs. (2)–(3) by a reduced coefficient of variation (CV) of IRIs from the Poisson value of 1:

(5) $$\mathrm{CV}=\frac{\sigma_{T}}{\langle T\rangle }=\frac{\sqrt{1+{\left(p_{0}r\tau_{D}\right)}^{2}}}{1+p_{0}r\tau_{D}},$$

which has a minimum value of $1/\sqrt{2}$ at $p_{0}r\tau_{D}=1$ and a maximum value of 1 as $r\tau_{D}\to 0$ or $r\tau_{D}\to \infty$. For example, in the curves shown in Figs. 1c1–1c2, CV=0.82 at 2 Hz and 0.87 at 50 Hz.

### 2.2 Distribution of Release Times for a Poisson Spike Train Through Stochastic Facilitating Synapses

For facilitating synapses, we take the following form for release probability, $P_{\mathrm{rel}}(t)=p_{0}F(t)$, where between presynaptic spikes:

(6) $$\tau_{F}\frac{dF}{dt}=(1-F).$$

Each spike produces an increase in *F* from $F^{-}$ (which determines the release probability of that spike) to $F^{+}$ (which is the new release probability for an immediate, subsequent spike) such that

(7) $$F^{+}=F^{-}+f_{F}\left(\frac{1}{p_{0}}-F^{-}\right)=F^{-}+f_{F}\left(F_{\max}-F^{-}\right),$$

where $f_{F}$ is the facilitation factor, taking a value between 0 and 1, indicating the fractional increase from the pre-spike release probability toward a saturating release probability of $p_{0}F=1$.

To calculate the distribution of interrelease intervals (IRIs) we need to calculate the probability of release as a function of time, following a prior release. Although presynaptic spikes arrive with constant probability per unit time in a Poisson process, vesicle release occurs more often when the facilitation variable is high. Thus, immediately after release, the likelihood of release is greater than on average, because the facilitation variable takes some time (on the order of $\tau_{F}$) to return to a baseline value. Furthermore, when calculating the IRI distribution, we must be aware that $\langle F_{R}^{\infty }(r)\rangle$, which is the mean value approached by *F* conditioned on no intervening release event will be lower than the mean value, $\langle F^{-}\rangle$, since long IRIs are more associated with time windows of fewer intervening presynaptic spikes than chance.

To proceed, we first calculate $\langle F_{R}^{+}(r)\rangle$, the mean of the facilitation variable immediately after vesicle release. To arrive at this quantity, we use the mean value of the facilitation variable averaged across all presynaptic spikes [17]:

(8) $$\langle F^{-}\rangle =\frac{\left[1+r\tau_{F}f_{F}/p_{0}\right]}{1+f_{F}r\tau_{F}}$$

and the variance of this quantity [9]:

(9) $$\sigma_{F^{-}}^{2}=\frac{r\tau_{F}f_{F}^{2}{\left(1/p_{0}-1\right)}^{2}}{{\left(1+r\tau_{F}f_{F}\right)}^{2}[2+r\tau_{F}f_{F}(2-f_{F})]}.$$

Together, these can be used to calculate $\langle F_{R}^{-}(r)\rangle$, which is the mean value of the facilitation variable just prior to firing when averaged across only those spikes that actually cause release, since release probability is proportional to $F^{-}$. The latter averaging produces a higher value than $\langle F^{-}\rangle$, since higher instances of $F^{-}$ are more likely to result in release, so weight the average more than lower values:

(10) $$P\left(F_{R}^{-}\right)=\frac{F^{-}P(F^{-})}{\int_{0}^{\infty }F^{-}P(F^{-})dF^{-}}=\frac{F^{-}P(F^{-})}{\langle F^{-}\rangle },$$

where $P(F^{-})$ is the probability that *F* takes the value $F^{-}$ immediately prior to an incoming spike and the denominator normalizes the distribution. Hence,

(11) $$\langle F_{R}^{-}(r)\rangle =\int_{0}^{\infty }\frac{{\left(F^{-}\right)}^{2}P(F^{-})}{\langle F^{-}\rangle }=\langle F^{-}\rangle +\frac{\sigma_{F^{-}}^{2}}{\langle F^{-}\rangle }.$$

From this, the mean value of the facilitation variable immediately following vesicle release can be calculated as

(12) $$\langle F_{R}^{+}(r)\rangle =\langle F_{R}^{-}(r)\rangle (1-f_{F})+\frac{f_{F}}{p_{0}}=\left(\langle F^{-}\rangle +\frac{\sigma_{F^{-}}^{2}}{\langle F^{-}\rangle }\right)(1-f_{F})+\frac{f_{F}}{p_{0}}.$$

The above formula is exact and was matched by simulated data at all values of *r* simulated (data not shown).

To estimate the steady state value of *F* a long time from any prior release—a steady state that may never be reached if the product of firing rate and base release probability is much higher than $1/\tau_{F}$—we solve a self-consistency equation for this value, $\langle F_{R}^{\infty }(r)\rangle$ and ignore fluctuations by assuming release probability is $p_{0}\langle F_{R}^{\infty }(r)\rangle$ for each presynaptic spike. One can calculate then the probability of *N* spikes in a given interval, *T*, conditioned on the requirement that none of those spikes caused vesicle release, while the facilitation variable is at its mean steady state value of $\langle F_{R}^{\infty }(r)\rangle$. The result is:

(13) $$\begin{array}{rl} P(N,T|\mathrm{No}\;\mathrm{release}) & =\frac{P(N,T){\left(1-p_{0}\langle F_{R}^{\infty }(r)\rangle \right)}^{N}}{\sum_{N=0}^{\infty }P(N,T){\left(1-p_{0}\langle F_{R}^{\infty }(r)\rangle \right)}^{N}}\\ & =\frac{e^{-rT}{\left(rT\right)}^{N}{\left(1-p_{0}\langle F_{R}^{\infty }(r)\rangle \right)}^{N}/N!}{\sum_{N=0}^{\infty }e^{-rT}{\left(rT\right)}^{N}{\left(1-p_{0}\langle F_{R}^{\infty }(r)\rangle \right)}^{N}/N!}\\ & =e^{-rT(1-p_{0}\langle F_{R}^{\infty }(r)\rangle )}{\left[rT\left(1-p_{0}\langle F_{R}^{\infty }(r)\rangle \right)\right]}^{N}/N! \end{array}$$

which is the result for a Poisson process of modified rate, $r(1-p_{0}\langle F_{R}^{\infty }(r)\rangle )$. This allows us to self-consistently calculate $\langle F_{R}^{\infty }(r)\rangle$ by using the result for the mean value of the facilitation variable given such a modified spike rate, such that

(14) $$\langle F_{R}^{\infty }(r)\rangle \approx \frac{1+r(1-p_{0}\langle F_{R}^{\infty }(r)\rangle )\tau_{F}f_{F}/p_{0}}{1+r(1-p_{0}\langle F_{R}^{\infty }(r)\rangle )\tau_{F}f_{F}},$$

which can be solved using the quadratic formula to give

(15) $$\langle F_{R}^{\infty }(r)\rangle \approx \frac{2r\tau_{F}f_{F}+1-\sqrt{{\left(2r\tau_{F}f_{F}+1\right)}^{2}-4p_{0}r\tau_{F}f_{F}}}{2p_{0}r\tau_{F}f_{F}},$$

a value which is always below $\langle F^{-}\rangle$ and in close agreement with simulated data (not shown).

Finally, to fit the IRI distribution, we assumed exponential decay from $\langle F_{R}^{+}(r)\rangle$ to $\langle F_{R}^{\infty }(r)\rangle$ with a time constant such that the initial slope (when the probability of any intervening spikes is zero) matches that of an exponential decay to 1 with time constant $\tau_{F}$ (the initial rate of decrease of *F* in the absence of intervening spikes). That is, we take the release to follow an inhomogeneous Poisson process with a rate, which depends on time, *T*, since the prior release event, given by

(16) $$r_{R}(T)=p_{0}r\left\{\langle F_{R}^{\infty }(r)\rangle +\left[\langle F_{R}^{+}(r)\rangle -\langle F_{R}^{\infty }(r)\rangle \right]e^{-T/\tau_{\mathrm{eff}}(r)}\right\},$$

where

(17) $$\tau_{\mathrm{eff}}(r)=\tau_{F}\frac{\langle F_{R}^{+}(r)\rangle -\langle F_{R}^{\infty }(r)\rangle }{\langle F_{R}^{+}(r)\rangle -1}.$$

**Fig. 2:**
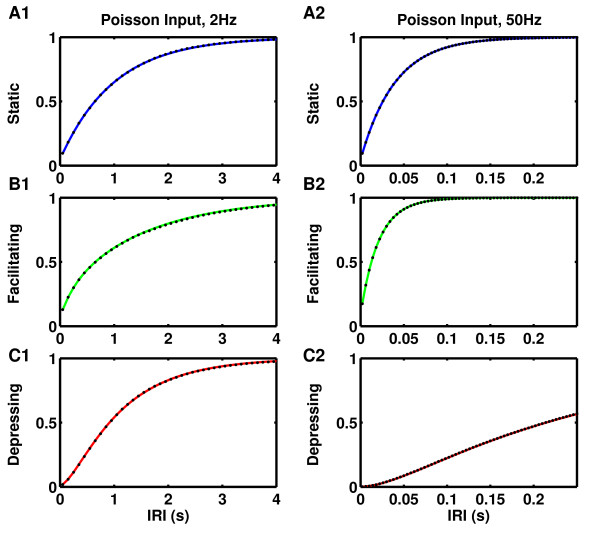
Cumulative distribution of inter-release intervals (IRIs) for the same curves shown in Fig. 1, verifying the remarkable agreement of the approximation used in Fig. 1b with the simulated data. **a1**–**a2** Static synapses, $p_{0}=0.5$. **b1**–**b2** Facilitating synapses, $p_{0}=0.1$, $f_{F}=0.5$, $\tau_{F}=500\text{ ms}$. **c1**–**c2** depressing synapses, $p_{0}=0.5$, $\tau_{D}=250\text{ ms}$. **a1**, **b1**, **c1** Presynaptic Poisson spike train of 2 Hz. **a2**, **b2**, **c2** Presynaptic Poisson spike train of 50 Hz

The distribution of IRIs is then given by [17]

(18) $$P(T)=r_{R}(T)\exp\left[-\int_{0}^{T}r_{R}(t)dt\right]$$

a function, which is plotted in Figs. 1b1–1b2, where it is indistinguishable from the simulated data. Similarly, indistinguishable is the cumulative IRI distribution plotted in Figs. 2b1–2b2, justifying the approximations that led to our results. 

Finally, it should be noted that when synapses are facilitating, consecutive IRIs are correlated. For example, when the presynaptic rate is 2 Hz in the simulation used to produce Figs. 1b1 and 2b1, the correlation between one IRI and the subsequent one is 0.028, while with a presynaptic rate of 50 Hz the correlation is 0.015. Such a correlation, which cannot be obtained from the IRI distribution alone, further increases any variability in postsynaptic conductance, above and beyond the increase due to the altered shape of the IRI distribution.

In summary, the main difference produced by facilitation from the exponential distribution of inter-spike intervals (which is retrieved by setting either $f_{F}$ or $\tau_{F}$ to zero) is an enhancement of probability at low *T* and a corresponding reduction at high *T*. These changes produce a CV of IRIs greater than 1 (CV=1.18 at 5 Hz and CV=1.03 at 50 Hz in the examples shown in Figs. 1b1–1b2) enhancing the noise in any neural system.

### 2.3 Mean Synaptic Transmission via Dynamic Synapses

We assume that at the time of vesicle release the postsynaptic conductance increases in a step-wise manner, with a fraction, $\tilde{\alpha }$, of previously closed channels becoming opened. This causes the synaptic gating variable, *s*, to increase from its prior value, $s^{-}$ to $s^{+}$ according to $s^{+}=s^{-}+\tilde{\alpha }(1-s^{-})$. It then decays between release events with time constant, $\tau_{s}$, according to $\tau_{s}\frac{ds}{dt}=-s$.

**Fig. 3:**
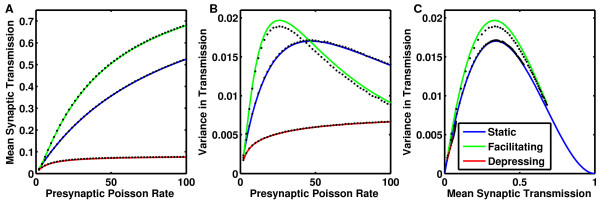
**a** Mean synaptic transmission, 〈s〉 and **b** variance of synaptic transmission, $\langle s^{2}\rangle -{\langle s\rangle }^{2}$, arising from presynaptic Poisson trains through probabilistic synapses. **c** Variance in synaptic transmission as a function of the mean transmission. *Solid curves* are analytic solutions (*blue, middle curve* for static, **a**= Eq. (23), **b**= Eq. (24), **c**= Eq. (25); *green, upper curve* for facilitating, **a** from Eqs. (20) and (31), **b** from Eqs. (22) and (31), **c** from **a** &**b**; and *red, lower curve* for depressing synapses, **a**= Eq. (26), **b** from Eqs. (4), (22), (25) and (27), **c** from **a** &**b**). *Black dots* are corresponding results from simulations produced by 30,000 sec of Poisson input spike trains through saturating synapses

If one assumes that successive inter-release intervals (IRIs) are uncorrelated then one can calculate the mean, 〈s〉, and variance, $\sigma_{s}^{2}=\langle s^{2}\rangle -{\langle s\rangle }^{2}$, in the postsynaptic gating variable (and hence the postsynaptic conductance, which is proportional to *s*) via:

(19a) $$\langle s^{-}\rangle =\langle s^{+}\rangle \langle e^{-T/\tau_{s}}\rangle ,$$

(19b) $$\langle s^{+}\rangle =\langle s^{-}\rangle (1-\tilde{\alpha })+\tilde{\alpha },$$

(19c) $$\langle s\rangle =\frac{\tau_{s}}{\langle T\rangle }\langle s^{+}\rangle \left(1-\langle e^{-T/\tau_{s}}\rangle \right)=\frac{\tau_{s}}{\langle T\rangle }\left(\langle s^{+}\rangle -\langle s^{-}\rangle \right),$$

where the averages of $\langle e^{-T/\tau_{s}}\rangle$ and 〈T〉 are taken over the distribution of interrelease intervals, P(T), as given in the prior section (Fig. 1, Eqs. (3), (18)) and we have used the solution $s(t)=s^{+}e^{-(t-t_{i})/\tau_{s}}$ at time *t* following the *i*th spike at time $t_{i}$. Solution of the above equations leads to

(20) $$\langle s\rangle =\frac{\tilde{\alpha }\tau_{s}}{\langle T\rangle }\frac{\left(1-\langle e^{-T/\tau_{s}}\rangle \right)}{\left[1-(1-\tilde{\alpha })\langle e^{-T/\tau_{s}}\rangle \right]},$$

which allows us to calculate the mean synaptic conductance through static and dynamic synapses (Fig. 3a). 

Similarly, combining

(21a) $$\langle {\left(s^{-}\right)}^{2}\rangle =\langle {\left(s^{+}\right)}^{2}\rangle \langle e^{-2T/\tau_{s}}\rangle ,$$

(21b) $$\langle {\left(s^{+}\right)}^{2}\rangle =\langle {\left(s^{-}\right)}^{2}\rangle {\left(1-\tilde{\alpha }\right)}^{2}+2\tilde{\alpha }(1-\tilde{\alpha })\langle s^{-}\rangle +{\tilde{\alpha }}^{2},$$

(21c) $$\langle s^{2}\rangle =\frac{\tau_{s}}{2\langle T\rangle }\langle {\left(s^{+}\right)}^{2}\rangle \left(1-\langle e^{-2T/\tau_{s}}\rangle \right),$$

leads to

(22) $$\langle s^{2}\rangle =\frac{\tau_{s}{\tilde{\alpha }}^{2}}{2\langle T\rangle }\frac{\left(1-\langle e^{-2T/\tau_{s}}\rangle )[(1-\tilde{\alpha })\langle e^{-T/\tau_{s}}\rangle +1\right]}{\left[1-{\left(1-\tilde{\alpha }\right)}^{2}\langle e^{-2T/\tau_{s}}\rangle ][1-(1-\tilde{\alpha })\langle e^{-T/\tau_{s}}\rangle \right]},$$

which allows us to calculate the variance in postsynaptic conductance (Fig. 3b).

When synapses are static, release times are distributed as a Poisson process of rate $rp_{0}$, where *r* is the presynaptic Poisson rate and $p_{0}$ is the static release probability. In this case, the mean value of the gating variable is calculated by standard methods [20] to give

(23) $$\langle s_{\mathrm{Static}}\rangle =\frac{\tilde{\alpha }\langle P_{\mathrm{rel}}r\rangle \tau_{s}}{1+\tilde{\alpha }\langle P_{\mathrm{rel}}r\rangle \tau_{s}}=\frac{\tilde{\alpha }p_{0}r\tau_{s}}{1+\tilde{\alpha }p_{0}r\tau_{s}},$$

a function plotted in Fig. 3a (blue curve), where it exactly matches the simulated data (black asterisks). A similar calculation leads to the variance in synaptic transmission for static synapses [12,20] as

(24) $$\sigma_{s,\mathrm{Static}}^{2}=\langle s_{\mathrm{Static}}^{2}\rangle -{\langle s_{\mathrm{Static}}\rangle }^{2}=\frac{{\tilde{\alpha }}^{2}p_{0}r\tau_{s}[1+(2-\tilde{\alpha })p_{0}r\tau_{s}]}{\left(1+\tilde{\alpha }p_{0}r\tau_{s})[2+\tilde{\alpha }p_{0}r\tau_{s}(2-\tilde{\alpha })\right]}-{\langle s_{\mathrm{Static}}\rangle }^{2},$$

a function plotted in Fig. 3b (blue curve), where it exactly matches the simulated data (black asterisks). The variance can be written as a function of the mean synaptic transmission by substituting for *r* into Eq. (24) with $\langle s_{\mathrm{Static}}\rangle$ from Eq. (23) to produce the reduced formula:

(25) $$\sigma_{s,\mathrm{Static}}^{2}=\frac{\tilde{\alpha }\langle s_{\mathrm{Static}}\rangle }{2-\tilde{\alpha }\langle s_{\mathrm{Static}}\rangle }{\left(1-\langle s_{\mathrm{Static}}\rangle \right)}^{2},$$

which is plotted in Fig. 3c (blue curve).

For probabilistic depressing synapses with “all-or-none” release, the IRIs are independent as the synapse is always in the same state immediately post-release. The IRIs are distributed according to Eq. (3), which leads to

(26) $$\langle e^{-T/\tau_{s}}\rangle =\frac{r\tau_{s}^{2}}{\left(\tau_{s}+\tau_{D})(1+r\tau_{s}\right)}$$

so that using Eq. (4) for the mean IRI, 〈T〉, we have

(27) $$\langle s_{\mathrm{Depress}}\rangle =\frac{\tilde{\alpha }p_{0}r\tau_{s}(\tau_{s}+\tau_{D}+p_{0}r\tau_{s}\tau_{D})}{\left(1+p_{0}r\tau_{D})(\tau_{s}+\tau_{D}+p_{0}r\tau_{s}\tau_{D}+\tilde{\alpha }p_{0}r\tau_{s}^{2}\right)},$$

which, plotted as a red curve in Fig. 3a, precisely matches the simulated data (black circles). Similarly, making the substitution for probabilistic depressing synapses:

(28) $$\langle e^{-2T/\tau_{s}}\rangle =\frac{r\tau_{s}^{2}}{\left(\tau_{s}+2\tau_{D})(2+r\tau_{s}\right)}$$

into Eq. (22), allows us to evaluate $\langle s_{\mathrm{Depress}}^{2}\rangle$ as plotted in Fig. 3b (red solid curve), where it precisely matches the simulated data (black points).

For probabilistic facilitating synapses, we use an approximate formula for P(T) to evaluate the expected value of the exponential decay $\langle e^{-T/\tau_{s}}\rangle$—essentially a Laplace transform—since the full formula is intractable for these purposes. We found after testing many formulas against simulated quantities that so long as we correctly included the facilitation factor immediately after release as $\langle F_{R}^{+}(r)\rangle$ and the approximate release probability a long time after release as $\langle F_{R}^{\infty }(r)\rangle$, the principal requirement was to use a probability density of IRIs, with the correct value for the mean IRI. For facilitating synapses we know the mean IRI, 〈T〉:

(29) $$\langle T\rangle =\frac{1}{p_{0}\langle F^{-}\rangle r}.$$

We fulfilled these three requirements by grossly simplifying the actual decay of the facilitation variable post-release, letting it switch between its immediate post-release value of $\langle F_{R}^{+}(r)\rangle$ to its steady state value, $\langle F_{R}^{\infty }(r)\rangle$, at a time, $T^{*}$ into the IRI where $T^{*}$ is chosen to produce the correct value of 〈T〉. That is, we approximated the probability distribution of IRIs, P(T), as

(30) $$\begin{array}{rl} P\left(T|T<T^{*}\right) & =rp_{0}\langle F_{R}^{+}(r)\rangle e^{-rp_{0}\langle F_{R}^{+}(r)\rangle T},\\ P\left(T|T\geq T^{*}\right) & =rp_{0}\langle F_{R}^{\infty }(r)\rangle e^{-rp_{0}\langle F_{R}^{\infty }(r)\rangle T}, \end{array}$$

where

(31) $$T^{*}=\frac{-1}{rp_{0}\langle F_{R}^{+}(r)\rangle }\ln\left(\frac{1/\langle F^{-}\rangle -1/\langle F_{R}^{+}(r)\rangle }{1/\langle F_{R}^{\infty }(r)\rangle -1/\langle F_{R}^{+}(r)\rangle }\right).$$

From such a distribution we can easily calculate moments, $\langle e^{-T/\tau_{s}}\rangle$ and $\langle e^{-2T/\tau_{s}}\rangle$, of the postsynaptic conductance using the Laplace transforms where

(32) $$\begin{array}{rl} \langle e^{-T/\tau_{s}}\rangle = & \frac{rp_{0}\langle F_{R}^{+}(r)\rangle \tau_{s}}{1+rp_{0}\langle F_{R}^{+}(r)\rangle \tau_{s}}\\ & +e^{-T^{*}/\tau_{s}}e^{-rp_{0}\langle F_{R}^{+}(r)\rangle T^{*}}\\ & \times \left(\frac{rp_{0}\langle F_{R}^{\infty }(r)\rangle \tau_{s}}{1+rp_{0}\langle F_{R}^{\infty }(r)\rangle \tau_{s}}-\frac{rp_{0}\langle F_{R}^{+}(r)\rangle \tau_{s}}{1+rp_{0}\langle F_{R}^{+}(r)\rangle \tau_{s}}\right). \end{array}$$

**Fig. 4:**
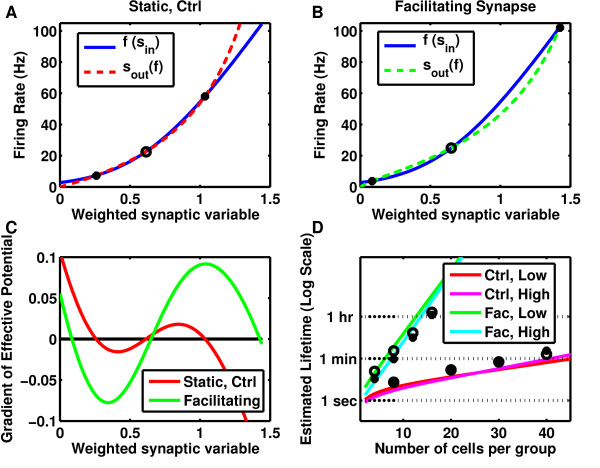
Synaptic facilitation enhances the stability of discrete memory states. **a** The firing rate curve (*solid, blue*) and synaptic feedback (*dashed red*) for a system with feedback strength optimized for bistability in a group of cells with static synapses. Firing rate curve follows Eq. (34) with $\beta_{1}=119.5$, $\beta_{2}=0.615$, $\beta_{3}=5.326$, which is the best fit to the leaky-integrate and fire neuron used in the simulations and described in Table 2d. Feedback strength is optimized for bistability with W=1.84. **b** Same firing rate curve (*solid, blue*) as in **a** but synaptic feedback (*dashed green*) via facilitating synapses with feedback strength optimized for bistability with W=2.10. **a**–**b** Solid circles indicate stable fixed points separated by an unstable fixed point (*open circle*). **c** Difference between firing rate and feedback curves in **a** and **b** determine the basis for the gradient of an effective potential. Note the enhanced areas between fixed points (*zero crossings*) producing a larger potential barrier when synapses are facilitating (*green*) compared to static (*red*). **d** The lifetime of both the low activity state and the high activity state increases exponentially with system size, but a given level of stability is achieved with far fewer cells when the synapses are facilitating (*solid curves*, analytic results; *filled and open circles* simulated results for the high and low activity states, respectively)

The corresponding mean postsynaptic conductance, using Eq. (20), plotted in Fig. 3a (green curve) is indistinguishable from the simulated data (black points). This form of the mean synaptic transmission through facilitating synapses will be used in the next section when we assess the stability and robustness of memory states produced by such synaptic feedback. The variance in synaptic transmission of the simulated data (Fig. 3b, black points) is no longer precisely fit by the approximate formula, obtained from Eq. (22), Eq. (31) and using Eq. (31) with $\tau_{s}$ replaced by $\tau_{s}/2$ to calculate $\langle e^{-2T/\tau_{s}}\rangle$ (Fig. 3b, green curve). However, since the approximate formula slightly overestimates the variance, it will tend to underestimate the stability of any memory state. Thus, a more precise fit would enhance stability (Fig. 4d). Figure 3c (green curve) indicates that for all values of mean synaptic transmission, the variance is greater when synapses are facilitating. 

## 3 Stability of Discrete States Enhanced by Short-Term Synaptic Facilitation

Groups of cells with sufficient recurrent excitatory feedback can become bistable, capable of remaining, in the absence of input, in a quiescent state of low-firing rate, or after transient excitation, in a persistent state of high-firing rate. Given the inherent stochastic noise in neural activity—spike trains are irregular, with the CV of ISIs often exceeding one—the activity states have an inherent average lifetime, which increases exponentially with the number of neurons in the cell-group. In this section, we show analytically that addition of synaptic facilitation to all recurrent synapses can increase the stability of such discrete memory states by many orders of magnitude. We follow the methods presented in a prior paper for static synapses [12] and extend them to a circuit with probabilistic facilitating synapses. Calculations of stability are based on the mean of first-passage times between two stable states [21]. We assume that neurons spike with Poisson statistics, while the variability in the postsynaptic conductance, which possesses a long time constant (100 ms) typical of NMDA receptors [15], determines the instability of states. Since synaptic facilitation of probabilistic synapses affects both the mean and variance of the postsynaptic conductance (Figs. 3a–3b), both must be calculated and taken into account when determining the lifetime of memory states. We describe the method briefly below, leaving a reproduction of the full details to the following sections.

Bistability arises when the deterministic dynamics of the network produces multiple fixed points—firing rates at which dr/dt=0—at least two of which are stable. The deterministic mean firing rate depends on the total synaptic input to a group of cells. The total synaptic input includes a feedback component via recurrent connections as well as an independent external component. At a fixed point, the feedback produced by a given firing rate is such that the total synaptic input exactly maintains that given firing rate (intersections in Figs. 4a, 4b). For a network to possess multiple fixed points, the curve representing synaptic transmission as a function of firing rate and the curve representing firing rate as a function of synaptic input must intersect at multiple points (Figs. 4a, 4b). Between any two stable fixed points is an unstable fixed point, where the curves cross back in the opposite direction. The stability of any individual fixed point is strongly dependent on the area enclosed between the two curves from that fixed point to the unstable fixed point. This enclosed area acts as the height of an effective potential (Fig. 4c), which, for a given level of noise in the system determines the mean passage time from one stable fixed point to the basin of attraction of the other fixed point, i.e., the mean lifetime of the memory state. Importantly, the lifetime is approximately exponentially dependent on the effective barrier height, or the area between the two curves. Thus, changes in the curvature of synaptic feedback as a function of firing rate, which can have a strong impact on the area between the f-I curve and the feedback curve, can affect state lifetimes exponentially.

When we analyze the extent of this effect as wrought by synaptic facilitation, we find a greatly enhanced barrier in the effective potential (Fig. 4c), which demonstrates the additional curvature in the neural feedback function outweighs any increase in noise in the system (which enters the denominator in the effective potential, Eq. (35). Consequently, the lifetime of both persistent and spontaneous states in a discrete attractor system, can be enhanced by several orders of magnitude when synapses are facilitating (Fig. 4d). Alternatively, one can obtain the same necessary stability with far fewer cells, for example, to produce a mean stable lifetime of over a minute for both the low and high activity states, with all-to-all connections, only eight cells are necessary in the example with facilitating synapses, whereas forty are necessary when synapses are static.

### 3.1 Analytic Calculation of Mean Transition Time Between Discrete Attractor States

To calculate transition times between discrete attractor states, and hence assess their stability to noise, we produce an effective potential for the postsynaptic conductance as the most slowly varying continuous variable of relevance. We use standard methods for transitions between stable states of Markov processes [21] but first must calculate the deterministic term, A(s), and diffusive term, D(s), for a group of cells with recurrent feedback. The calculations in the case of static synapses were produced and validated elsewhere [12] but we briefly reiterate them in the following paragraphs. When synapses are facilitating, the only alterations are the expression for mean synaptic conductance, 〈s(r)〉 (Fig. 3a) and its variance, $\sigma_{s(r)}^{2}$ (Fig. 3b), and a newly optimized strength of feedback connection to ensure both spontaneous and active states remain as stable as possible.

Our essential assumption is to treat the behavior of the postsynaptic variable, *s*, given a presynaptic Poisson spike train at rate *r*, as an Ornstein–Uhlenbeck process, which matches the mean and variance of *s*, while maintaining the same basic synaptic time constant for decay to zero in the absence of presynaptic input. Thus, we have

(33) $$A(s)=-\frac{s}{\tau_{s}}+\frac{\tilde{\alpha }}{\langle T\rangle }-\frac{\tilde{\alpha }s}{\tau_{s}}\frac{\langle e^{-T/\tau_{s}}\rangle }{\left(1-\langle e^{-T/\tau_{s}}\rangle \right)},$$

(by matching the mean of *s*) and

(34) $$D_{1}(s)=-2\sigma_{s}^{2}\frac{dA(s)}{ds}$$

(by matching the variance of *s*) where the subscript “1” indicates the variance produced by a single presynaptic spike train. For a circuit with *N* presynaptic neurons producing feedback current, we scale down individual connections strengths so that the mean feedback current is independent of *N*, but the noise is reduced as $D_{N}(s)=D_{1}(s)/N$, since *s* is the fraction of maximal conductance (0≤s≤1).

We close the feedback loop by ensuring the presynaptic firing rate is equal to the postsynaptic firing rate, so use the firing rate function [22]:

(35) $$r=f(S)=\frac{\beta_{1}(S-\beta_{2})}{1-\exp[-\beta_{3}(S-\beta_{2})]},$$

with rate multiplier $\beta_{1}=115$, threshold $\beta_{2}=0.571$, and concavity $\beta_{3}=5.66$ all obtained by fitting to leaky integrate-and-fire simulations [12]. *S* is a scaled version of *s*, accounting for the total feedback conductance, S=Ws, where *W* is the sum of connection strengths of all cells and held fixed when *N* is varied.

The effective potential, Φ(s), for a group with *N* feedback inputs per cell is

(36) $$\Phi (s)=-2N\int_{0}^{s}\frac{A(s^{\prime })}{D_{1}(s^{\prime })}ds^{\prime },$$

which leads to a probability density, P(s):

(37) $$P(s)=\frac{2N}{CD_{1}(s)}\exp\left[-\Phi (s)\right],$$

where *C* is a normalization constant. The mean transition time from a stable state centered at $s_{1}$ to a state centered at $s_{2}>s_{1}$ is [21]:

(38) $$T_{\mathrm{trans}}(s_{1},s_{2})=\frac{C}{2}\int_{s1}^{s_{2}}ds\exp\left[\Phi (s)\right]\int_{0}^{s}P\left(s^{\prime }\right)ds^{\prime },$$

a function which is plotted for both static and facilitating synapses in Fig. 4d.

### 3.2 Simulation of Mean Transition Time Between Discrete Attractor States

We compared the results of our approximate analysis (Fig. 4d, curves) with those of computer simulations of noisy leaky-integrate and fire neurons. To do this, we simulated small circuits of excitatory neurons connected in an all-to-all manner, using the parameters given in Table 2. Each neuron received independent background Poisson inputs, both excitatory and inhibitory, such that interspike intervals had a CV of 1 at low firing rates, decreasing gradually to 0.8 by a firing rate of 100 Hz. We simulated for either 200,000 seconds, or until 20,000 transitions between states were made, whichever was sooner. The mean transition times are plotted in Fig. 4d (open and closed circles), where they show good qualitative agreement with the analytic curves. 

**Table 2:** Details of network simulations producing memory activity

(a) Model summary	
Populations	Single population, E
Connectivity	All-to-all
Neuron model	Leaky integrate-and-fire (LIF) with refractory period
Synapse model	Excitatory AMPA + voltage-dependent NMDA, inhibitory GABA conductances − step increase then exponential decay
Input	Independent fixed-rate Poisson spike trains from populations of Input cells
Measurements	State transitions times via mean population firing rate

(b) Populations		
Name	Elements	Size
E	LIF neurons	*N* = 8,20,30,40 (static) *N* = 4,8,12,16 (facilitating)

(c) Connectivity			
Name	Source	Target	Pattern
EE	E	E	All-to-all, weight *W*

(d) Neuron and synapse model	
Name	LIF neuron
Type	Leaky integrate-and-fire (LIF) with refractory period, and noisy Poisson exponential conductance input
Subthreshold dynamics	$C_{m}\frac{dV}{dt}=g_{L}(V-V_{L})+g_{\mathrm{EE}}(t)(V-V_{E})+g_{\mathrm{AMPA}}(t)(V-V_{E})+g_{\mathrm{GABA}}(t)(V-V_{I})$
EE synaptic conductance dynamics	$g_{\mathrm{EE}}(t)=g_{\mathrm{EE}}^{\max}\sum_{\mathrm{cells},i}s_{i}(t)$
	$\tau_{s}\frac{ds_{i}}{dt}=-s_{i}(t)$ between spikes of cell *i* at times $t_{i}^{*}$ and $s_{i}(t_{i}^{*+})=s_{i}(t_{i}^{*-})+\tilde{\alpha }[1-s_{i}(t_{i}^{*-})]$
Spiking	If $V(t)>V_{th}$ then (1) emit spike with time-stamp $t^{*}$ (2) $V(t)\to V_{\mathrm{reset}}$

(f) Input	
Type	Description
Poisson generators *X* = AMPA,GABA	$\tau_{X}\frac{ds_{X}}{dt}=-s_{X}(t)+\sum \delta (t-t_{X}^{*})$
	$P(t\leq t_{X}^{*}<t+dt)=\upsilon_{X}dt$; $\upsilon_{\mathrm{AMPA}}=\upsilon_{\mathrm{GABA}}=800\text{ Hz}$

(g) Measurements	
Transition times	Time for $\langle s_{i,\mathrm{EE}}\rangle$ to transition from below 0.05 to above 0.45 ($T_{\mathrm{down}}$) and from above 0.45 to below 0.05 ($T_{\mathrm{up}}$)

(h) LIF neuron parameters											
$V_{L}$	$V_{E}$	$V_{I}$	$V_{th}$	$V_{\mathrm{reset}}$	$g_{L}$	$g_{\mathrm{EE}}^{\max}$	$C_{m}$	$g_{\mathrm{AMPA}}^{\max}$	$g_{\mathrm{GABA}}^{\max}$	$\tau_{\mathrm{AMPA}}$	$\tau_{\mathrm{GABA}}$
−70 mV	−70 mV	−70 mV	−45 mV	−60 mV	50 nS	$\frac{30\;\mathrm{nS}}{N}$	0.5 nF	20 nS	20 nS	2 ms	5 ms

(i) Synaptic parameters (EE)					
Synapse	Presynaptic *τ*	$p_{0}$	Factors	Postsynaptic $\tau_{s}$	$\tilde{\alpha }$
Static	–	0.5 (M)	–	100 ms	1 − exp(−0.25)
Facilitating	$\tau_{F}=500\;\mathrm{ms}$	0.25 (M)	$f_{F}=0.25$ (M)	100 ms	1 − exp(−0.25)

### 3.3 Results for Multiple Circuits

In the example shown, bistability in the control system with static synapses required particular fine-tuning of parameters, so was not very robust. One could wonder that if a different system were chosen—in particular a different f-I curve were used—then the system with static synapses might not be improved by the addition of synaptic facilitation. That is, should synaptic facilitation always enhance robustness of such bistable neural circuits? To address this point, we parametrically varied the properties of the f-I curve (Eq. (34)) and for each set of parameters, $\left\{\beta_{1},\beta_{2},\beta_{3}\right\}$ we systematically varied the feedback connection strength, *W*, to test whether the system could be bistable.

**Fig. 5:**
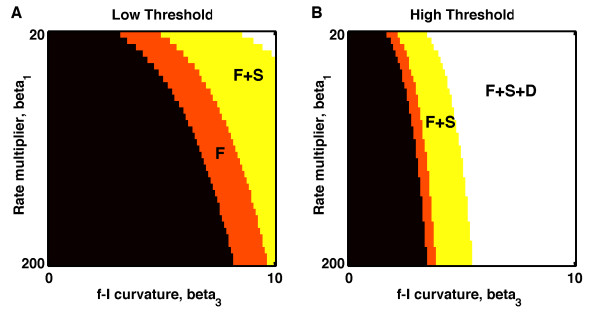
Range of bistability is enhanced with facilitating synapses and reduced with depressing synapses. **a** Low threshold, with $\beta_{2}=0.301$. **b** High threshold with $\beta_{2}=1.001$. **a**, **b***White region*, all models (with static, facilitating and depressing synapses) are bistable; *yellow region*, models with static or facilitating synapses are bistable; *orange region*, only models with facilitating synapses are bistable; *black region*, no models are bistable

As a result (Fig. 5), we found that the set of parameters $\left\{\beta_{1},\beta_{2},\beta_{3}\right\}$ able to produce bistability when synapses are static is a subset of the set found when synapses are facilitating. Thus, synaptic facilitation can produce bistability when it is not possible with static synapses, but the reverse is not true. As a corollary, the set of parameters $\left\{\beta_{1},\beta_{2},\beta_{3}\right\}$ able to produce bistability when synapses are depressing is a subset of the set found when synapses are static. 

**Fig. 6:**
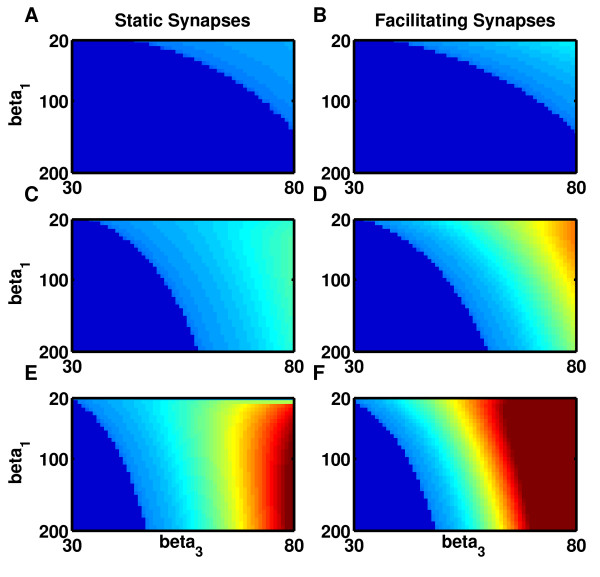
Maximum stability of memory states, for a given neural firing-rate curve, is always greater when synapses are facilitating rather than static. **a**–**b** Low threshold, $\beta_{2}=0.451$. **c**–**d** Medium threshold, $\beta_{2}=0.701$. **e**–**f** High threshold, $\beta_{2}=0.951$. **a**, **c**, **e** Synapses are static. **b**, **d**, **f** Synapses are facilitating. *All panels*: Steepness of single neuron firing-rate curves increase with $\beta_{1}$ (*y-axis*) while maximum curvature increases with $\beta_{3}$ (*x-axis*). Stability of a bistable system is determined by the minimum lifetime of either of the two activity states. Maximum stability is calculated for each firing-rate curve as a function of connection strength and plotted after logarithmic scaling in *color* code. *Dark blue*: no bistability exists. *Light blue* = low stability; *orange-red* = high stability; *cyan-green* boundary = optimal lifetime of one hour

For all parameter sets $\left\{\beta_{1},\beta_{2},\beta_{3}\right\}$ able to produce bistability, we assessed the optimal stability of the memory system. As the excitatory feedback connection strength, *W*, increases, so the mean lifetime of the high-activity state increases, while the mean lifetime of the low-activity state decreases. We consider optimal stability of the memory state as the value of the lifetime when high-activity and low-activity states are equally durable. More specifically, we calculate the minimum of $T_{\mathrm{trans}}(s_{1},s_{2})$ and $T_{\mathrm{trans}}(s_{2},s_{1})$ as a measure of the stability of memory and parametrically vary *W* to find the maximum stability for a given set of $\left\{\beta_{1},\beta_{2},\beta_{3}\right\}$ and given type of synapses. In all cases where comparison was possible, stability is enhanced when synapses are facilitating and stability is reduced when synapses are depressing, compared to the case of static synapses (Fig. 6). 

**Fig. 7:**
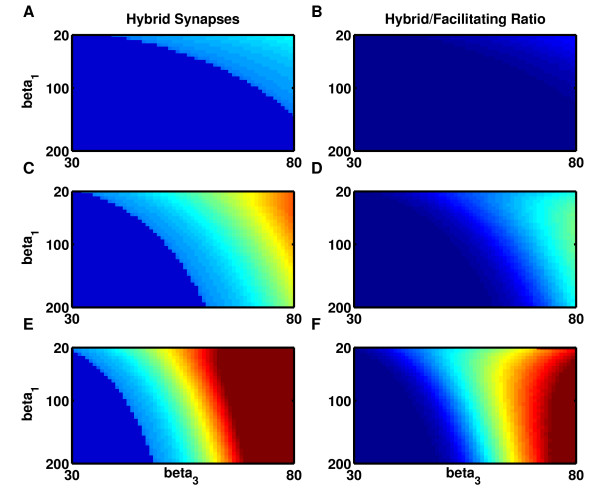
A hybrid model demonstrates the reduction in lifetime attributable to the enhanced fluctuations in postsynaptic conductance produced by synaptic facilitation. **a**–**b** Low threshold, $\beta_{2}=0.451$. **c**–**d** Medium threshold, $\beta_{2}=0.701$. **e**–**f** High threshold, $\beta_{2}=0.951$. **a**, **c**, **e** Lifetime of states in the hybrid model with synaptic facilitation but with the noise due to static synapses. *Dark blue*: no bistability exists. *Light blue* = low stability; *orange-red* = high stability; *cyan-green* boundary = optimal lifetime of one hour. **b**, **d**, **f** Logarithm of the ratio of Figs. 7a, 7c, 7e to 6b, 6d, 6f, respectively, demonstrates the decrease in state lifetime attributable to enhanced noise when synapses are facilitating. *Dark blue*: ratio = 1. *Cyan-yellow*, ratio >*e*. *Orange-light red*, ratio $>e^{2}$. *Dark red*, ratio $>e^{3}$. *All panels*: Steepness of single neuron firing-rate curves increase with $\beta_{1}$ (*y-axis*) while maximum curvature increases with $\beta_{3}$ (*x-axis*). Stability of a bistable system is determined by the minimum lifetime of either of the two activity states. Maximum stability is calculated for each firing-rate curve as a function of connection strength and plotted after logarithmic scaling in color code

It is worth emphasizing that the two effects of synaptic facilitation on synaptic transmission have opposing consequences for attractor state stability. While the increased curvature in the curve of mean synaptic transmission increases stability of discrete attractors, the increased variance (Fig. 3c, green curve) decreases stability. While our results demonstrate that the deterministic effect dominates (i.e., the net effect of facilitation is to enhance stability), it is instructive to assess the contribution of each of the two effects alone. Thus, for a given mean synaptic transmission calculated for facilitating synapses, we used the variance in synaptic transmission corresponding to static synapses (Fig. 3c, blue curve) and recalculated the lifetimes of memory states. While changing the noise does not change significantly the parameter range for bistability (i.e., Fig. 5 is, to first order, unaffected by changes in noise) it does have a considerable impact on the lifetimes of states. In particular, by using the reduced noise of static synapses—a reduction of at most 20 %—the optimal lifetime was typically a factor of *e* higher in a circuit with 20 neurons and $e^{2}$ higher in a circuit with 40 neurons (using the parameters of Fig. 4d). Figure 7 demonstrates the enhanced lifetime in the hybrid model across networks—the ratio is always greater than one and extended to as high as 50 in the networks examined. Thus, the increased noise in the postsynaptic current produced by synaptic facilitation does produce considerable destabilization of state lifetimes—the hybrid model of synaptic facilitation without such enhanced noise produces the greatest possible stability of discrete memory states. 

## 4 Discussion

Bistability relies upon positive feedback, which can arise from cell-intrinsic currents or from network feedback. Synaptic facilitation is a positive feedback mechanism in circuits of reciprocally connected excitatory cells, since the greater the mean firing rate, the greater the effective connection strength, further amplifying the excitatory input beyond that produced by the increased spike rate alone. This property of synaptic facilitation enhances the stability of memory states and renders them more robust to distractors [23]. Other forms of positive feedback, such as depolarization-induced suppression of inhibition (DSI), which depends on activity in the postsynaptic cell, can similarly produce robustness in recurrent memory networks [24]. 

When the bistability necessary for discrete memory is produced through synaptic feedback in a circuit of neurons, the relative stability to noise fluctuations of each of the two stable fixed points depends exponentially on the area between the mean neural response curve and the synaptic feedback curve (Figs. 4a–4b). While the synaptic feedback curve is monotonic in firing rate, for static synapses it is either linear (in the absence of postsynaptic saturation) or of negative curvature (decreasing gradient), with the effectiveness of additional spikes decreasing at high rates when receptors become saturated. However, when the synapse is facilitating, the synaptic response curve has positive curvature when firing rates are low—the effect of each additional spike is greater as firing rate increases. Here, we showed how such an effect could increase the area between intersections of synaptic feedback and neural response curves, enhancing stability dramatically (Figs. 4–6).

We note that the addition of positive curvature at low rates to the negative curvature at high, saturating rates in the curve of synaptic transmission as a function of presynaptic firing rates (Fig. 3a) inevitably increases the areas between three points of intersection with *any* firing rate curve without such an “S”-shape (Figs. 4a–4b). Since the “S”-shape is a hallmark of synaptic facilitation, not present for synaptic transmission through static synapses, facilitation can always enhance stability of such bistable systems. Less mathematically, a facilitating synapse with the same effective strength as a static synapse at intermediate firing rates is stronger at high firing rates, enhancing the stability of a high-activity state (where a drop in synaptic transmission is detrimental), while at the same time is weaker at low firing rates, enhancing the stability of a low-activity state (where a rise of synaptic transmission is detrimental).

It is worth pointing out the converse—that short-term synaptic depression reduces the robustness of such discrete attractors. Indeed, in Fig. 5, we show that the range of parameters for which a bistable system exists is much narrower when synapses are depressing (D) versus static (S) or facilitating (F). Since synaptic depression contributes a negative curvature to the f-I curve, it tends to reduce the “S-shape” needed for bistability. Or, perhaps more intuitively, high synaptic strength is needed to maintain a high-firing rate state if synapses are depressing, but such high synaptic strength is more likely to render the low-firing rate spontaneous state unstable.

The changes in the shape of the distribution of inter-release intervals caused by dynamic synapses alter the fluctuations in post-synaptic conductance. In particular, facilitation enhances the variability and depression reduces the variability arising from a Poisson spike train. While the extra variability caused by facilitating synapses tends to destabilize a memory system, this effect was overwhelmed by the increase in stability due to the rate-dependent changes in mean synaptic transmission described above. However, the increase in conductance variability, in particular, being on a slower timescale than membrane potential fluctuations, can be a factor in explaining the high CV of neural spike trains.

Our calculations are based on a simplified formalism, in which the firing-rate curve (f-I curve) of a neuron is first assumed or fit (Eq. (34), [22]) under in vivo-like conditions, assuming a given level of noise in the membrane potential. Since the shape of the f-I curve depends on both the mean and variance of the input current [25,26], it might appear invalid to discuss changes in the variability of input current due to dynamic synapses in the context of a fixed f-I curve. However, the time constants for short-term synaptic plasticity and the NMDA receptor-mediated currents are more than an order of magnitude greater than the time constant of the membrane potential under the conditions of strong, fluctuating balanced input that produce the irregularity of spike trains seen in vivo. Since the neuron’s membrane potential can sample its probability distribution—which determines the likelihood of a spike per unit time—more rapidly than the timescale for changes in that probability distribution, our analytic methods provide a reasonable description of the circuit’s behavior (Fig. 4d).

In summary, we have demonstrated the ability of short-term synaptic facilitation to stabilize discrete attractor states of neural activity to noise. We have shown this by simulations and through analytic methods, which include a consideration of how stochastic dynamic synapses mold the distribution of interrelease intervals (IRIs) into a form that differs from the exponential distribution of incoming interspike intervals (ISIs). The altered IRI distribution affects both mean synaptic transmission and the variability of transmission due to a presynaptic Poisson spike train—both of which have a strong impact on the stability of memory states. The increased variability of synaptic transmission due to facilitation is more than countered by the effect of facilitation on mean synaptic transmission, which enhances the robustness of bistability, leading to stable memory states with fewer neurons.

## Competing Interests

The author declares that he has no competing interest.
